# Alterations in Salivary Proteome following Single Twenty-Minute Session of Yogic Breathing

**DOI:** 10.1155/2015/376029

**Published:** 2015-03-19

**Authors:** Sundaravadivel Balasubramanian, Michael G. Janech, Graham W. Warren

**Affiliations:** ^1^Department of Radiation Oncology, Hollings Cancer Center, Medical University of South Carolina, Charleston, SC 29425, USA; ^2^Division of Nephrology, Department of Medicine, Medical University of South Carolina, Charleston, SC 29425, USA; ^3^Department of Cell and Molecular Pharmacology, Medical University of South Carolina, Charleston, SC 29425, USA

## Abstract

Yogic breathing (YB) has been suggested to reduce stress and blood pressure and increase cognitive processes. However, alterations after YB at the molecular level are not well established. Twenty healthy volunteers were randomized into two groups (*N* = 10 per group): YB or attention controls (AC). The YB group performed two YB exercises, each for ten minutes, for a total of twenty minutes in a single session. AC group read a text of their choice for 20 minutes. Saliva was collected at baseline and at 5, 10, 15, and 20 minutes. Using Mass Spectrometry (MS), we initially found that 22 proteins were differentially expressed and then validated deleted in malignant brain tumor-1 (DMBT1) and Ig lambda-2 chain C region (IGLC2) using Western Blotting. DMBT1 was elevated in 7 of YB group by 10-fold and 11-fold at 10 and 15 minutes, respectively, whereas it was undetectable in the time-matched AC group (*P* < 0.05). There was a significant interaction between groups and time assessed by two-way ANOVA (*P* < 0.001). IGLC2 also showed a significant increase in YB group as measured by Western Blotting. These data are the first to demonstrate the feasibility of stimulating and detecting salivary protein biomarkers in response to an acute Yoga exercise. This trial is registered with ClincalTrials.gov NCT02108769.

## 1. Introduction

Cultural practices have long played an important role in human health. Incorporated into daily routines, food habits, ethics, sports, social activities, religious ceremonies, and festivities, these practices are considered to promote the overall well-being of individuals belonging to that cultural group [1]. Yoga is the collection of numerous mind-body techniques from the ancient Eastern cultural practices with the main theme of unification (the Tamil word “*okka*” or the Sanskrit word “*yok*” means to unite or equalize). Although practiced for its claimed benefits of healthy living and stress relief, the molecular mechanisms underlying how Yoga could improve health are only beginning to emerge. Yogic breathing (YB, also called* Pranayamam* or* Pranayama*) is one of the Yoga practices and is an active way of regulating breathing. Thirumoolar, a saint from ancient times, wrote Thirumanthiram, a Tamil literary work containing several Yogic and Tantric methods [2, 3]. There are 14 songs in Thirumanthiram specifically on Yogic breathing (verses 564–577). Although Yoga practitioners widely practice Pranayama techniques, the techniques of Thirumoolar have not yet been studied for their biological effects or molecular changes in biomarkers. Earlier physiological studies with other breathing regulation methods suggest that reducing the breathing frequency (around 15/min in normal adults [4]) could reduce blood pressure among heart failure patients [5]. As Pranayama leads to predominance in abdominal/diaphragmatic breathing [5–7], it increases vagal tone and parasympathetic dominance and decreases sympathetic discharges [8, 9].

Chanting Om is another type of YB, also called* Pranava Pranayama*. Chanting Om is an ancient cultural practice believed to improve physical and mental health. Early stage investigations on* Pranava Pranayama* suggest that it could (a) reduce heart rate and blood pressure in hypertensive patients [10], (b) promote physical and emotional well-being [11, 12], (c) increase cutaneous peripheral vascular resistance [12], (e) induce vagal nerve stimulation (VNS) [13, 14], and (f) deactivate the limbic brain regions, amygdala, hippocampus, parahippocampal gyrus, insula, and orbitofrontal and anterior cingulate cortices and thalamus [12]. However, most if not all these studies are pilot in nature and therefore the results have to be validated for elucidating biological mechanism.

In this line recent studies have begun to unravel the molecular mechanisms of Yoga and other similar practices. For instance, in response to meditation, Black et al. reported the possible involvement of transcriptional regulation in peripheral blood lymphocytes indicative of overall reduction of stress response [15]. Similarly, Bhasin et al. have shown that Relaxation Response including Yoga, meditation, and repetitive prayer seems to improve mitochondrial resiliency by increasing the gene expression of ATPase and insulin function, while decreasing the gene expression of NF-*κ*B associated stress response genes among practitioners [16]. Changes in gene expression following 2 hours of comprehensive Yoga practice involving postures, breathing, and meditation indicated significant change in gene expression in immune response genes in peripheral blood mononuclear cells [17]. Bower et al. have demonstrated a significant reduction in interferon-related transcription factors and NF-*κ*B targets following 12-week Yoga intervention in breast cancer survivors [18]. These studies suggest that Yoga practices could potentially alter the expression of genes associated with inflammation and stress response. However, these studies have relied upon blood as the major source of biomarkers to study gene expression, and proteome level changes were not measured following Yoga practice. Moreover, the molecular mechanisms of Pranayama in isolation have not yet been studied in detail. Currently there are no established protein biomarkers to help measure the effects of YB on clinical outcomes or well-being. Identification of useful biomarkers would significantly increase the ability of differentiating objective from subjective responses reported by patients or participants in a study. Saliva is an easily accessed biological sample that contains numerous biomarkers including proteins, peptides, metabolites, mRNA, DNA, and miRNA of both human and oral microbial origin [19–22]. Due to the noninvasive nature and relative ease of sample collection, saliva has been increasingly recognized as a rich source of biomarkers useful in many diseases. For instance, salivary proteomic and mRNA profiling have identified significant differences between control and oral cancer subjects [23].

As salivation is one of the parasympathetic activation responses [24], we hypothesized that Pranayama might activate salivation and that the proteomic profile of saliva thus produced will be different from the basal saliva. Our initial mass spectrometry (MS) analysis revealed changes in the levels of 22 proteins following YB. To validate our MS data by Western Blotting, we chose the protein candidates deleted in malignant brain tumor 1 (DMBT1) and Ig lambda-2 chain C region (IGLC2) based on their abundance in saliva, spectral counts and level of statistical significance in MS data, and the roles of these proteins in immune regulation, epithelial differentiation, tumor suppression, and stress response [25, 26].

## 2. Methods

### 2.1. Human Subjects

A total of twenty healthy volunteers (male or female), aged 18 and above, were included in the study. The exclusion criteria were as follows: breathing problems (inability to breathe through nostrils, chronic bronchitis, emphysema, and asthma), speech problems that would prevent chanting, inability to listen and follow study exercise, sinus congestion, Sjogren's syndrome, chronic dry mouth due to medication or other conditions, and use of anticholinergic medications. Informed consent was obtained from each subject after initial interview. The study protocol was approved by the Institutional Review Board for Human Research, Medical University of South Carolina. Participants were enrolled after informed written consent. Recruitment of participants was carried out in Charleston Metro area from August 15, 2013, to October 31, 2013. The protocol requires the participant to attend only one 20-minute session with no follow-up. This study is registered at the ClinicalTrials.gov. This study was not registered prior to enrollment of participants owing to the small number of participants required for the protocol. The authors confirm that all ongoing and related trials for this drug/intervention are registered.

**Figure 1:**
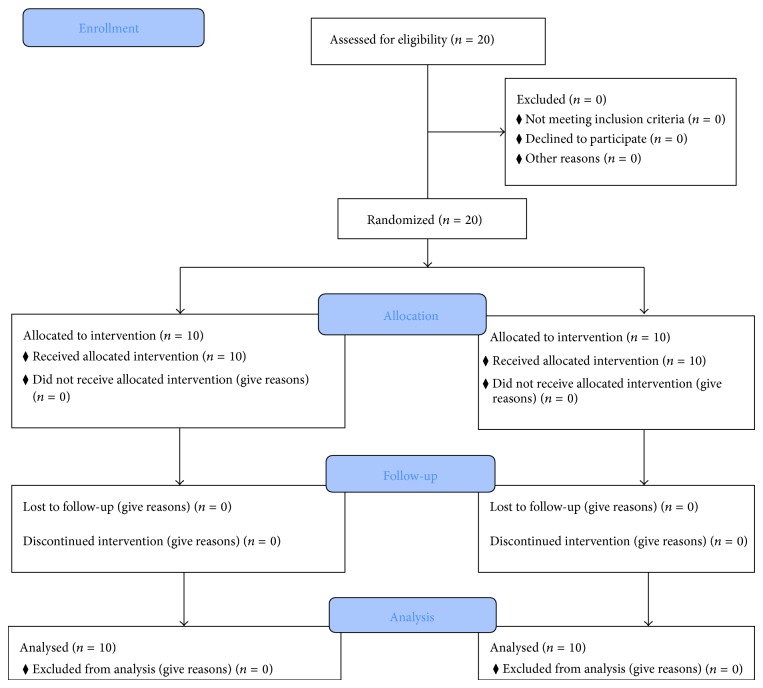
CONSORT flowchart. Details of the overall trial design.

Enrolled participants were randomized to one of 2 conditions: Yogic breathing (YB) arm versus the Attention Control (AC) arm (see Figure 1 for CONSORT flowchart). Randomization was conducted in collaboration with a biostatistician to ensure equal gender distribution in the 2 experimental groups (YB versus AC). All the participants were tested one-on-one with a trained Yoga instructor. Prior to exercise and sample collection, the Yoga instructor taught each subject how to perform YB.

## 3. Treatment Conditions

### 3.1. Yogic Breathing

**Figure 2:**
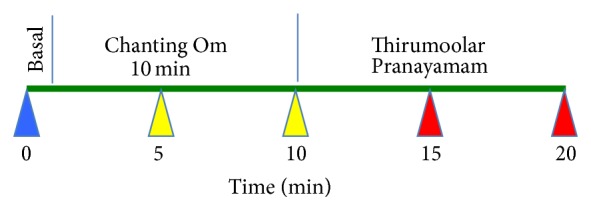
Yogic breathing intervention. Yogic breathing exercise contains two phases, namely, Chanting Om and Thirumoolar Pranayamam, each for 10 minutes. Saliva sample is collected starting from 0 min and every five minutes as shown.

The YB exercise design is depicted in Figure 2. The study Yoga instructor taught the subjects how to perform the YB exercises, which consist of Om Chanting and Thirumoolar Pranayamam as detailed below.

#### 3.1.1. Chanting Om

The subjects were seated in a chair with eyes closed while chanting. The subjects performed Chanting Om as follows: (a) long deep inhalation through both nostrils at the same time and (b) slow exhalation while chanting “Om.” These two steps were repeated continuously for 10 min with a brief interruption at 5 minutes to collect saliva. Saliva was immediately placed on ice after collection.

#### 3.1.2. Thirumoolar Pranayamam (TP)

Following the chanting, the subjects performed TP as follows, as instructed by the Yoga instructor based on Thirumanthiram (verse 568) [2, 3]: During TP, the inhalation/holding/exhalation cycles each lasting in seconds were counted as follows using the combination of chanting and counting with fingers. Repeatedly chant a phrase within mind (e.g., “*I'm beautiful,*” “*I'm relaxed,*” “*Om Namasivaya,*” etc.) for two times (inhalation), eight times (holding), and four times (exhalation). (a) Close right nostril and inhale through left nostril for two chants and then close both nostrils so that no inhaled air escapes. (b) Hold breath in this position for eight chants mentally. (c) Open right nostril and exhale for four chants. Complete exhalation is required. (d) Go to step (a) and repeat. The subjects performed TP for 10 min. Salivary samples were collected at 5 and 10 minutes of TP (see below). Thus each individual provided the following five saliva samples: basal (0 min), Chanting Om (5, 10 min), and TP (15, 20 min).

### 3.2. Attention Control Group

Attention Control subjects performed quiet reading for 20 min in a seated position in the presence of the Yoga instructor. Saliva samples were collected at 0, 5, 10, 15, and 20 min similar to the YB group.

### 3.3. Collection of Saliva Samples

Salivary samples were collected once at the beginning of the protocol (Time 0) while the subjects are seated. Samples were subsequently collected at 5, 10, 15, and 20 min from both groups of participants. Saliva was naturally allowed to accumulate in the oral cavity and the participant discharged (1–4 mL) into the 15 mL polystyrene specimen tube with lid. Samples were immediately cooled on ice and stored at −80°C until analysis within 15 minutes of collection.

### 3.4. Liquid Chromatography-Tandem Mass Spectrometry (LC-MS-MS)

For initial LC-MS-MS analysis we used saliva samples from a single individual, the Yoga instructor and the author of this paper. The saliva sample was collected before and after 20 minutes of YB exercise done on six different days. Protein concentration in raw saliva was determined using the Bio-Rad protein assay based on the Bradford method (Bio-Rad, Hercules, CA). For each sample, 100 *μ*gs protein was diluted in 100 *μ*Ls mass spectrometry grade water. To the diluted protein, 100 *μ*Ls ammonium bicarbonate (100 mM) was added and the sample was vortexed. Proteins were reduced using 5 mM dithiothreitol and heated to 60°C for 30 minutes. Samples were allowed to cool to room temperature and proteins were alkylated using 12 mM iodoacetamide at room temperature for 30 minutes. Trypsin (Trypsin Gold, Promega) was added to each sample at a ratio of 1 : 50 and incubated at 37°C for 18 hours. Samples were acidified using formic acid to a final concentration to 0.1% and peptides were passed over a conditioned solid phase cartridge (Strata-X, Phenomenex, 60 mg/mL). Peptides were washed with 0.1% formic acid and eluted in 50% acetonitrile/0.1% formic acid. Peptides were dried by centrifugal vacuum and resuspended in 0.1% formic acid. Peptide concentration was estimated by absorbance at 280 nm and all samples were diluted with equal volumes of 0.1% formic acid to a final concentration of 0.4 *μ*g/*μ*L.

Peptides (4 *μ*g) were trapped onto an Acclaim PepMap100 trap column (100 *μ*m ID × 2 cm, C18, 5 *μ*m, 100 Å, Thermo Scientific) using 100% mobile phase A (MPA, 98% water, 0.1% formic acid, 2% acetonitrile) at a flow rate of 5 *μ*L/min, for 5 minutes. Trapped peptides were separated on an Acclaim PepMap100 analytical column (75 *μ*m ID × 15 cm, C18, 3 *μ*m, 100 Å, Thermo Scientific) using an Eksigent NanoLC Ultra System. A gradient of mobile phase B (MPB, 2% water, 0.1% formic acid, 95% acetonitrile) to MPA was increased from 5% to 40% over 40 minutes and then increased from 40% to 80% over 10 minutes. Peptides were introduced into a nanosource and tandem mass spectrometry was performed using an AB SCIEX Triple TOF 5600 mass spectrometer (ABSCIEX, Framingham, MA). Data were collected in information dependent acquisition mode with the following parameters: 250-millisecond MS accumulation time, 100-millisecond product ion accumulation time, 20-ion selected per cycle, total cycle time of 1.3 seconds, charge states for selection = +2 to +4, 6-second exclusion after one occurrence, and rolling collision energy. The scanning windows for the TOF-MS and product ion scans were 300 to 1250 and 100 to 1600 *m*/*z*, respectively. Time of flight collision energy was set to 10 and declustering potential was set to 100.

### 3.5. Protein Identification and Quantification

Acquired raw data (.wiff) was converted to  .MGF format using the AB SCIEX converter (version 1.2 beta). MGF files were searched against the 2012_1 release of the Human UniProtKB/Swiss-Prot database (37,303 entries) with addition of proteins from the common Repository of Adventitious Proteins database (cRAP; 2012.01.01; the Global Proteome Machine, 112 entries) using the Mascot search engine. The following parameters were selected: Digestion Enzyme, semitrypsin; parent ion tolerance, 20 ppm; fragment ion tolerance, 0.25 Da; fixed modification, carbamidomethyl(C); variable modification, pyro-Glu(Q), oxidation(M). Mascot search results were uploaded to Scaffold (version 3.1.2, Proteome Software Inc., Portland, OR) and scored by Peptide Prophet and Protein Prophet algorithms. Peptide threshold was set to 80%, protein threshold was set to 99.9%, and minimum 2 peptides were required resulting in a false-discovery rate equal to 0.8%. Protein spectral counts were normalized to total spectral counts using the “quantitative value” tool. Spectral count values of 0 were given a minimum value of 1. The gene ontology tool was utilized in Scaffold to categorize proteins.

### 3.6. Western Blotting

Western Blotting was performed as previously described [27, 28]. Briefly, 100 *μ*L of saliva samples was mixed with equal volume of 2X SDS-sample buffer and heated at 95°C for 5 min for denaturation. After centrifugation for 5 min at 14,000 rpm at room temperature (RT), 20 *μ*L of the denatured samples was resolved on 4–12% Bis-Tris gel with MOPS running buffer (Life Technologies) and transferred to PVDF membranes. Membranes were blocked with 2% BSA in Tris-buffered saline containing 0.05% Tween (TBST) for 1 h at RT and incubated with 1 : 1000 diluted primary antibodies: DMBT1 (sc-28239, Santa Cruz Biotechnology) and Ig lambda-2 chain C region (MAB219P, Maine Biotechnology Services) overnight at 4°C. After washing and incubation for 1 hour at RT with the secondary antibodies conjugated to horse radish peroxidase (1 : 10,000 dilution), the bands were visualized using enhanced chemiluminescence. The membrane was then stained with Ponceau S (Sigma) to determine protein loading. The scanned images of the Western Blots were quantified using NIH ImageJ. The DMBT1 protein levels were normalized to Ponceau S staining and represented as graph.

### 3.7. Statistical Analysis

Comparisons were performed between baseline and specific time points (5, 10, 15, and 20 min after YB). Pre- and postmeasurements were analyzed using a paired *t*-test because all comparisons passed normality testing (Shapiro-Wilk test). Differences were accepted when *P* < 0.05. For Western Blot analysis, a two-way repeated measures ANOVA was utilized. Values were log-transformed prior to statistical analysis. Pairwise post hoc comparisons were made using the Holm-Sidak test. Differences were accepted when *P* < 0.05.

## 4. Results

All the participants enrolled and randomized completed the trial. There is no dropout or adverse effects.

### 4.1. Discovery Proteomic Study

Label-free proteomics resulted in a total of 133 proteins identified at a protein false-discovery rate of 0.8%. Gene ontology (GO) annotation categorized 93 out of 133 (70%) proteins under the head node, biological process, in the selected GO term: cellular process. A little more than a quarter of the proteins within this head node (36/133, 27%) belonged to immune system process. The majority of proteins in the cellular compartment head node (74/133, 56%) were assigned to the extracellular region.

Comparison of proteins by normalized spectral counting revealed significant changes in 22 proteins (Table 1) following YB in a single participant who repeated the study Yogic Breathing exercises on six different days. Five proteins were lower in abundance and 17 proteins were elevated ranging from −1.8 to 3.8 mean fold change. The majority of statistically significant proteins (36%) belong to the immunoglobulin family and every member identified in this group was elevated.

### 4.2. Targeted Western Blots

**Figure 3:**
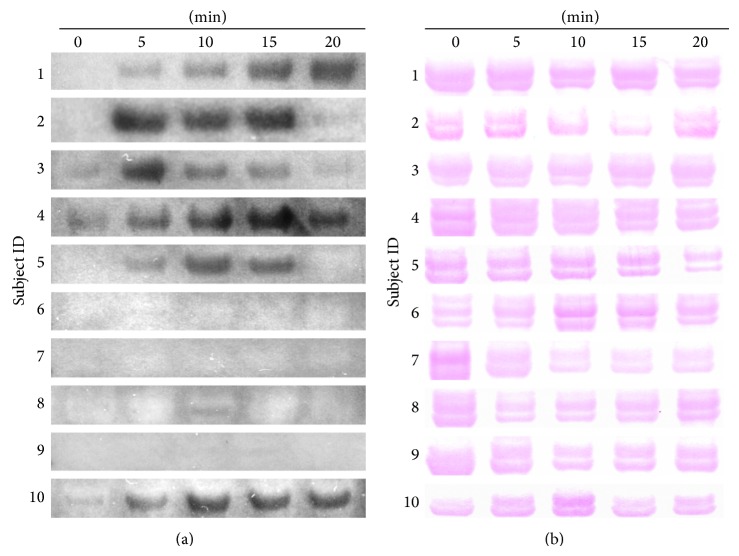
Increased salivary DMBT1 abundance in Yogic breathing participants. Saliva from Yogic breathing group participants (*n* = 10) was collected at indicated time points. Equal volumes of saliva were loaded and analyzed by Western Blotting with DMBT1 antibody as described under Methods. DMBT1 band around 220 kDa is shown (a). The same membrane was stained with Ponceau S and shown for loading control (b).

**Figure 4:**
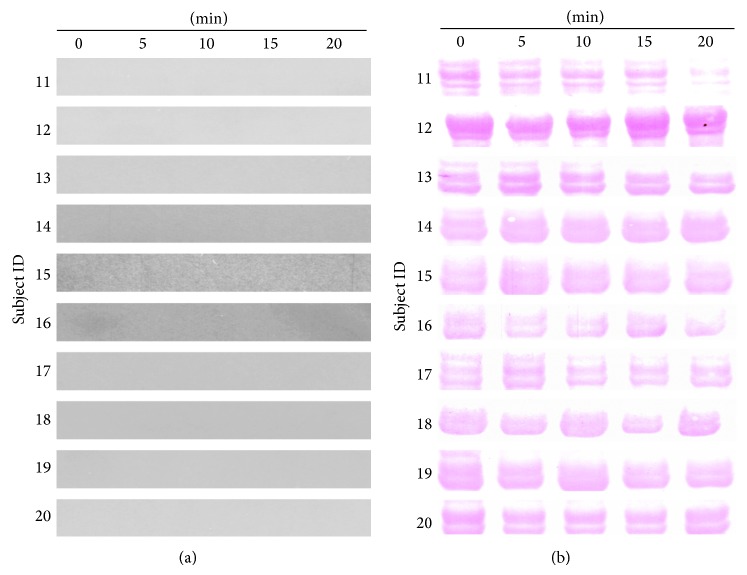
Undetectable salivary DMBT1 level in Attention Control participants. Saliva from Attention Control group participants (*n* = 10) was collected at indicated time points. Equal volumes of saliva were loaded and analyzed by Western Blotting with DMBT1 antibody as described under Methods. There was no DMBT1 band detected (a). The same membrane was stained with Ponceau S and shown for loading control (b).

Expression of salivary DMBT1 using Western Blot analysis is shown for all of the samples from YB group (Figure 3(a)) and AC group (Figure 4(a)). Elevated expression of DMBT1 was observed in 7 out of 10 subjects from the YB group and none in the AC group. Elevations in DMBT1 level were observed as early as 5 min and sustained through 15 min in all participants who demonstrated elevated DMBT1 following YB. In 4 of the 7 participants with elevated DMBT1, the increase in expression was reversed by 20 min following YB suggesting that the effects of YB on DMBT1 expression may be acute.

**Figure 5:**
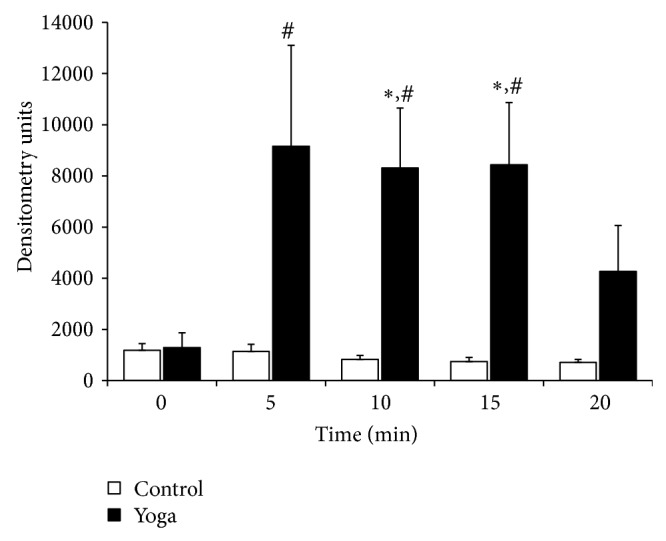
Pooled densitometry data of DMBT1 Western Blots. Protein abundance was quantified by densitometry from Western Blotting (Figures 3(a) and 4(a)) and data are presented as Mean ± Standard Error of relative units. ∗ indicates *P* < 0.05 control versus Yogic breathing group for each respective time point. # indicates *P* < 0.05 for Yogic breathing participants at the indicated time point versus time zero. Statistical differences were determined by two-way ANOVA with Holms-Sidak post hoc test.

Relative DMBT1 levels as determined by densitometry were different between AC and YB groups (Figure 5). There was a significant interaction between groups and time assessed by two-way ANOVA (*P* < 0.001). Both AC and YB groups were similar at baseline (0 min). DMBT1 was elevated by 10-fold and 11-fold at 10 and 15 minutes, respectively, in the YB group compared to the time-matched AC group (*P* < 0.05; Holm-Sidak pairwise comparison). Absolute elevations in DMBT1 expression by Western Blot were comparable in magnitude to elevations noted by LC-MS-MS.

**Figure 6:**
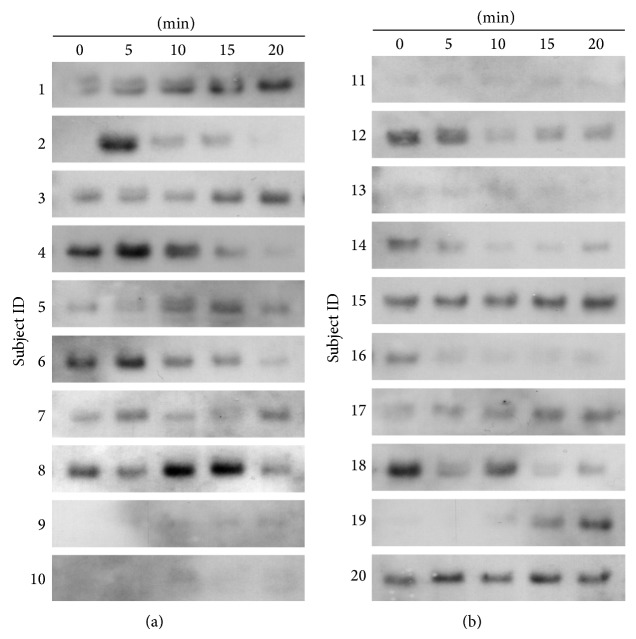
Level of IGLC2 in saliva from Yogic breathing and Attention Control groups. Saliva from Yogic breathing group (a) and Attention Control group (b) participants (*n* = 10 each) was collected at indicated time points. Equal volumes of saliva were loaded and analyzed by Western Blotting with IGLC2 antibody as described under Methods.

Further Western Blotting analysis on another candidate protein, IGLC2, with high spectral count in MS, fold change, and the level of statistical significance, showed a marked increase upon YB (Figure 6(a)). All of the 10 participants from the YB group showed an increase in IGLC2, where the overall level in two participants (numbers 9 and 10) was low. The increase was evident as early as 5 minutes. The level of IGLC2 returned to near basal by the end of 20 minutes of exercise except in two cases (numbers 1 and 3). This suggests that YB induces mainly a transient increase in IGLC2 level. It is interesting to note that, among AC subjects, two of them have a noticeable increase in IGLC2 level (numbers 15 and 19), whereas the rest of them did not show a market increase (Figure 6(b)). We further analyzed if Carbonic Anhydrase that did not have a significant change in mass spectrometry data could be useful as a loading control. However, due to variations within and between groups, we chose to use Ponceau S stain (Figures 3(b) and 4(b)) as loading control similar to previous studies [29]. Together, our data indicate that acute single session practice of Yogic Breathing when compared to Attention Control could stimulate alterations in salivary proteome.

## 5. Discussion

To the best of our knowledge this is the first time a salivary proteomic study has been applied to assess the physiological outcomes of Yogic Breathing. Yoga studies so far have relied mainly on subjective measures (e.g., quality of life questionnaire) and physiological outcomes such as blood pressure to determine the effectiveness of Yoga intervention. Recently, gene expression studies in peripheral blood following Yoga practice have identified a number of genes involved in metabolism, energy regulation, stress, and inflammation [15–18]. Saliva has been used only in the analysis of cortisol as a measure of stress in a recent Yoga study (6 wk Yoga with 1, 3, and 6 months of follow-up) among cancer patients [30]. We hypothesized that YB could acutely produce salivary biomolecules with key biological functions immediately after practice. We have recently reported that the study YB exercises induce nerve growth factor expression in saliva [31]. We expected that YB might alter the status of other biomarkers as well in saliva. Our initial LC-MS-MS analysis was conducted in a trained Yoga instructor before and after 20 min of YB regimen to identify potential markers that could then be analyzed in larger samples. Thus, our initial proteomic analysis showed a significant increase in the acute expression of molecules belonging to GO terms cellular processes, immune response, and those molecules were found mainly in the extracellular space. Of these molecules, several molecules belong to the Ig superfamily, suggesting that YB induces these molecules to promote immune response as a first line of defense in the mucosa. Interestingly, there were four tumor suppressors significantly stimulated after YB, namely, deleted in malignant brain tumor 1 (DMBT1), mucin-7 (MUC7), cysteine-rich secretory protein 3 (CRISP3), and prolactin-inducible protein (PIP). Notably all the above four proteins were found to be downregulated in a recent tumor suppressor gene analysis in oral squamous cell carcinoma using whole-genome analysis [32]. Based on the relatively well-defined multifunctional characteristics including immune regulation, epithelial differentiation, and tumor suppression in a number of tumors, we chose DMBT1 and IGLC2 to analyze further Yogic breathing samples obtained from the clinical trial. The expression data of DMBT1 and IGLC2 strongly indicate that YB significantly induces changes in these proteins in YB group when compared with AC group. The basal level of DMBT1 was different within YB group where only 3 of the YB group participants (numbers 3, 4, and 10) showed a detectable basal DMBT1 level whereas none in the AC group showed a detectable DMBT1 band. This could be due to several reasons including basal physiological variation of DMBT1 level or the psychological induction during randomization or other dietary and/or environmental factors that could not be accounted for in this study protocol.

DMBT1 is a product of* dmbt1* gene, which also codes for gp340/salivary agglutinin (SAG), an alternatively spliced variant from scavenger-receptor cysteine-rich (SRCR) superfamily. This gene also codes for a number of orthologs including Hensin, Ebnerin, CRP-ductin, and BGM/H3 [26]. DMBT1 is expressed in saliva, tears, breast milk, and gastrointestinal, genital, and pulmonary tract (reviewed in [33]). DMBT1 plays an important role in epithelial cell differentiation by binding to galectin-3. This binding is also considered important for activating the Notch signaling pathway and for the angiogenesis at the vascular extracellular matrix [34]. DMBT1 mediates innate immune response by binding to the bacterial and viral antigens. Binding of soluble DMBT1 is known to reduce the infectivity of HIV1 [35] as well as influenza A [36] viruses. DMBT1 has 14 SRCR domains and binds to a broad range of bacteria owing to the presence of common binding motif (RVELYxxxSW) within its SRCR domains [37]. DMBT1 is also shown to activate the complement pathway, further supporting its role in innate immune system [38].

In addition, DMBT1 is significantly downregulated in a number of cancers affecting multiple organs including brain, lungs, pancreas, oral cavity, prostate, cervix, and skin [26]. The reduced level of DMBT1 expression at the tumor site is attributed to the gene deletion of* dmbt1* in tumor cells. Incidentally,* dmbt1* gene is located within Chromosome 10q similar to PTEN, another tumor suppressor, implicating both of these molecules as important biomarkers in cancer prognosis. In fact,* dmbt1* is proposed as a breast cancer causative gene [39]. Stimulating salivary DMBT1 by a nonpharmacologic, noninvasive, behavioral intervention such as YB could hold several health benefits including maintenance of an effective innate immune system and production of tumor suppressors de novo. Although we have shown an increase of DMBT1 in saliva following YB, the possible mechanisms through which this happens are unknown. Signals emanating from extracellular matrix, epigenetic control (e.g., methylation [40]), and intracellular mechanisms could account for this induction. Moreover, as DMBT1 is a secretory protein, the increased level we observe in 5 minutes after YB could be due to the release from the secretory granules of ductal and acinar cells of the salivary glands.

Similarly, the increase in IGLC2 from our data is the first of its kind for this molecule to be tested in YB setting. IGLC2 level is increased in all the YB participants (although the level is low in two participants) but not in eight of the AC group participants. The reason why two participants from AC group have higher levels of IGLC2 is unclear. One could speculate that mere calming down (sitting and reading) might have an effect on IGCL2 in select participants. It is interesting to note that the majority of proteins (36%) that are significantly altered upon YB belong to the immunoglobulin superfamily. IGLC2 is encoded by chromosome 22 and is increased in several pathological conditions including HIV infection [41], multiple myeloma [42], and influenza A virus infection [43], suggesting that this induction could be involved in protection against infection and immune response. Although serum has higher kappa/lambda ratio, in mucosal secretions, such as saliva, this ratio is significantly reduced [25] suggesting the importance of IGLC2 in mucosal defense. Together, our data indicate that YB could increase salivary secretion that contains proteins with key roles in immune response. Further studies with large population are necessary to further validate these data. As one of the first studies in this area, our study has few limitations: the small number of subjects tested and that this is not a double-blinded study as the participants when informed that they are assigned to YB group might have had some elevation in basal DMBT1 due to psychological stimulation that is not related to the YB exercise per se. Mindful of these limitations, future studies could be focused, for example, to analyze if YB could stimulate DMBT1 in cancer patients and if YB could facilitate the availability of DMBT1 in tumor sites where DMBT1 is downregulated.

In conclusion, for the first time we are showing that an acute Yogic Breathing practice induces changes in levels of salivary proteins of significance to immune response and cancer. Future* Omic* studies using saliva as a source in individuals after both acute and long-term practice of various Yoga techniques could explain molecular mechanisms through which several Yoga techniques may work.

## Figures and Tables

**Table 1:** Differentially abundant proteins based on normalized spectral counts for the pre- and post-Yogic breathing samples. Protein identification was assigned by MASCOT search (FDR < 1%). Uniprot #: accession number from Uniprot for each protein. MW: predicted molecular weight. log_2_⁡FC: average ratio of normalized spectral counts after Yoga divided by those before Yoga for each respective time point. *P* value: probability value for paired *t*-test.

Protein identification	Uniprot #	MW	log_2_⁡FC	*P* value
Ig lambda-2 chain C regions	P0CG05	11 kDa	1.1	0.000
Mucin-7	Q8TAX7	39 kDa	0.8	0.002
Alpha-2-macroglobulin-like protein 1	A8K2U0	161 kDa	−1.4	0.006
Deleted in malignant brain tumors 1 protein	Q9UGM3	261 kDa	1.1	0.010
Immunoglobulin J chain	P01591	18 kDa	1.8	0.010
Ig alpha-1 chain C region	P01876	38 kDa	1.5	0.012
Ig mu chain C region	P01871	49 kDa	3.8	0.015
Ig heavy chain V-III region BRO	P01766	13 kDa	3.1	0.016
Cystatin-S	P01036	16 kDa	0.5	0.016
Keratin, type I cytoskeletal 10	P13645	59 kDa	−1.7	0.017
Prolactin-inducible protein	P12273	17 kDa	0.7	0.018
Ig alpha-2 chain C region	P01877	37 kDa	1.3	0.018
Keratin, type II cytoskeletal 5	P13647	62 kDa	−1.8	0.022
Glyceraldehyde-3-phosphate dehydrogenase	P04406	36 kDa	−1.1	0.020
Kallikrein-1	P06870	29 kDa	2.9	0.026
UPF0762 protein C6orf58	Q6P5S2	38 kDa	1.5	0.028
Ig kappa chain C region	P01834	12 kDa	1.0	0.029
Cystatin-B	P04080	11 kDa	−1.2	0.033
Ig heavy chain V-III region TUR	P01779	12 kDa	1.6	0.038
Cysteine-rich secretory protein 3	P54108	28 kDa	2.3	0.045
Zymogen granule protein 16 homolog B	Q96DA0	23 kDa	1.1	0.049
Keratin, type II cytoskeletal 6A	P02538	60 kDa	0.1	0.049
